# The pharmacokinetics of continuous subcutaneous levodopa/carbidopa infusion: Findings from the ND0612 clinical development program

**DOI:** 10.3389/fneur.2022.1036068

**Published:** 2022-11-10

**Authors:** Peter A. LeWitt, Fabrizio Stocchi, David Arkadir, Yoseph Caraco, Liat Adar, Itay Perlstein, Ryan Case, Nir Giladi

**Affiliations:** ^1^Department of Neurology, Wayne State University School of Medicine and Henry Ford Hospital, Detroit, MI, United States; ^2^Department of Neurology, University and Institute for Research and Medical Care Istituto di Ricovero e Cura a Carattere Scientifico (IRCCS) San Raffaele, Rome, Italy; ^3^Department of Neurology, The Faculty of Medicine, Hadassah Medical Center, Hebrew University of Jerusalem, Jerusalem, Israel; ^4^Clinical Pharmacology Unit, Division of Medicine, Hadassah Hebrew-University Medical Center, Jerusalem, Israel; ^5^NeuroDerm Ltd., Rehovot, Israel; ^6^Magic Wand Research, Philadelphia, PA, United States; ^7^Sackler School of Medicine, Tel Aviv Medical Center and Sagol School of Neurosciences, Neurological Institute, Tel-Aviv University, Tel Aviv-Yafo, Israel

**Keywords:** carbidopa, clinical development, levodopa, ND0612, Parkinson's disease, pharmacokinetics

## Abstract

**Background:**

While treatment with levodopa remains the cornerstone of Parkinson's disease (PD) management, chronic oral therapy is often associated with the development of motor complications, that correlate to fluctuating levodopa plasma concentrations, limiting its clinical utility. Continuous infusion is considered to be the optimal delivery route for treating PD patients with motor fluctuations, but current infusion systems require invasive surgery. Subcutaneous infusion of (SC) levodopa has the potential to provide a better tolerated and more convenient route of continuous levodopa delivery. ND0612 is in development as a combination product providing continuous levodopa/carbidopa *via* a minimally invasive, subcutaneous delivery system for PD patients experiencing motor response fluctuations. We present pharmacokinetic results from a series of studies that analyzed plasma concentrations after SC levodopa delivery with ND0612 to inform the clinical development program.

**Methods:**

We performed a series of six Phase I and II studies to characterize the pharmacokinetics of levodopa and carbidopa derived from ND0612 infusion with/without adjunct oral therapy of the same ingredients. These studies were conducted in healthy volunteers and in PD patients experiencing motor response fluctuations while on their current levodopa therapy regimen.

**Results:**

Taken together, the results demonstrate dose-proportionality dependent on rate of subcutaneous levodopa infusion leading to stable and sustained plasma concentrations of levodopa. Subcutaneous infusion of ND0612 administered with oral levodopa/carbidopa maintained near-constant, therapeutic levodopa plasma concentrations, thereby avoiding the troughs in levodopa plasma concentrations that are associated with OFF time in PD. The data generated in this series of studies also confirmed that a levodopa/carbidopa dose ratio of 8:1 would be the most reasonable choice for ND0612 development.

**Conclusions:**

This series of clinical pharmacokinetic studies have demonstrated that ND0612, administered continuously with a levodopa concentration of 60 mg/ml combined with carbidopa 7.5 mg/ml, and complemented with oral levodopa/carbidopa, is suitable for 24 h continuous administration in patients with PD. The stable plasma concentrations of levodopa achieved predict utility of ND0612 as a parenteral formulation for achieving clinically useful delivery of levodopa for PD patients.

## Introduction

Although more than two dozen drugs have undergone human testing as alternatives for achieving striatal dopaminergic stimulation, levodopa remains the most effective drug for controlling the motor symptoms of Parkinson's disease (PD) (1, 2). Most patients receiving levodopa chronically experience continuing benefits, although the consistency of antiparkinsonian effect from each oral dose tends to decline over time. A key driver in the search for improved therapies has been the problem of motor complications (response fluctuations and dyskinesia) that can develop with long-term levodopa use. Recent cohort studies estimate the 5-year cumulative incidence of response fluctuations and dyskinesias ranges between 29 and 54% in the overall levodopa-treated PD population (3–5), increasing to near-universal occurrence after 10 years or more of continuing levodopa use (5). These problems have significant impact on employment, independent functioning, safety, and quality of life (3, 6–10), particularly in patients with a younger age of onset (4). Response fluctuations evolve as patients experience the waning of the “long duration” response to levodopa dosing over time [the sustained motor improvement that builds up slowly and can persist for days after each dose (5)] and the “short duration” response (in which the therapeutic response closely parallels the plasma pharmacokinetics of levodopa) starts to predominate (11). The major pharmacodynamic challenge for optimizing PD therapeutics has been to reduce the variability of circulating levodopa concentrations to overcome the problems of the short duration response in order to lessen fluctuations in both motor and non-motor signs and symptoms.

Whereas, OFF states can have other causes to explain why PD patients are unresponsive to striatal dopaminergic stimulation (such as the phenomenon of gait freezing), the most common explanation for motor fluctuations is simply that of inconsistent levodopa delivery to the brain (11–13). Indeed, much of the blame for motor fluctuations falls on inconsistent gastrointestinal mechanisms leading to insufficient absorption of the drug, substantial intraindividual variability (14), and delayed onset of its antiparkinsonian effects (15, 16). Declining efficiency of the stomach to deliver oral levodopa to its sole absorption site in the proximal small intestine has been recognized as one of the consequences of chronic PD (16). Gastrointestinal dysfunction is frequent in PD, eventually affecting almost all patients during their disease course (16–18). For example, prolonged gastric emptying time can occur throughout the course of PD (18), and orally-administered levodopa can itself increase gastric acid secretion, impair gastric relaxation, and delay gastric emptying (19, 20).

Continuous levodopa delivery to the brain has not been possible to achieve with orally-dosed levodopa, even with pharmacological advances designed to achieve greater extension of levodopa release as compared to the immediate-release formulation (19). Drugs designed to block the peripheral catabolism of levodopa [catechol-O-methyltransferase (COMT) inhibitors] or retard the oxidative deamination of dopamine in the brain (monoamine oxidase-B inhibitors) achieve only a limited extension in duration of antiparkinsonian effect (21–23). When a patient has lost the long-duration response, the short plasma half-life of levodopa (~90–150 min) will require that immediate-release products be dosed repeatedly at intervals as close as 2–3 hourly intervals (22). Clinical experience shows that even these strategies, combined with MAO-B and COMT inhibitors, often fail to accomplish continuity of levodopa effect (24, 25). Thus, there has been a keen interest to improve levodopa therapeutics by optimizing its delivery to the brain by means of an alternative to its oral (and therefore gastrointestinal) delivery.

Pharmacodynamic studies exploring continuous intravenous levodopa infusion into a patient with PD first revealed the potential offered by parenteral levodopa delivery (26). Another route of administration has been developed in a marketed product for infusion of a carbidopa-levodopa suspension mixture continuously pumped into the proximal small intestine (27, 28). Partial reduction of OFF time and lessening of troublesome levodopa-induced dyskinesias has been accomplished by per-gastric intestinal infusion by the latter strategy. However, intra-intestinal delivery still delivers levodopa through the intestinal wall and has practical and tolerability issues that limit its use for the common problem of irregular dopaminergic effect (29, 30). One potential answer to the challenge of improving levodopa therapeutics is ND0612 (NeuroDerm, Rehovot, Israel), a combination product providing continuous levodopa/carbidopa *via* a minimally invasive, subcutaneous (SC) delivery system in development for people with PD experiencing motor fluctuations. As the first levodopa liquid formulation created for SC delivery, ND0612 has undergone a clinical development program with a series of early Phase I and II studies for characterizing and optimizing pharmacokinetics of its two key components, levodopa and carbidopa.

We present pharmacokinetic results from a series of Phase I and Phase II clinical studies that have analyzed plasma concentrations and pharmacodynamics of SC levodopa delivery. These studies informed the development of ND0612 as a PD therapy for patients with motor response fluctuations and have provided key information for its Phase III program. Clinical experience from these studies have provided insights into the pharmacokinetic behavior of levodopa and carbidopa when each are administered subcutaneously.

## Methods

### Study designs and participants

We performed a series of six Phase I and II studies to characterize the pharmacokinetics of levodopa and carbidopa derived from ND0612 infusion with/without adjunct oral therapy of the same active ingredients. These studies were conducted in healthy volunteers and in PD patients experiencing an established pattern of motor fluctuations while on their current regimen of levodopa and an inhibitor of aromatic L-amino acid decarboxylase, also known as dopa-decarboxylase (DDC), either carbidopa or benserazide. Table 1 provides an overview of the five study methodologies and Supplementary Table 1 provides further details per study. Each study was conducted in accordance with the Declaration of Helsinki and International Conference on Harmonization Good Clinical Practice Guidelines. All participants provided written informed consent. Institutional review boards at the participating sites approved the study protocol, consent forms, and associated amendments.

**Table 1:** Study methodologies.

**Study**	**Objective**	**Study design**	**Participant type (*N*)**	**Dosing & administration methods**
ND0612-001	Demonstrate stable plasma levodopa concentrations following ND0612 SC delivery in healthy volunteers	Phase I, single dose (24-h), single-center, randomized, double-blind, placebo-controlled dose escalation study (N=36)	36 healthy male volunteers (aged 18–40 years old)	• 24-h SC infusion of ND0612 (levodopa/carbidopa, 60/14 mg/ml) in one infusion site and placebo on the opposite side of the abdomen. • Doses of ND0612 were administered sequentially to cohorts of 6 volunteers. • Infusion rates: 80, 120, 160, and 200 h (short 6 mm and regular 10 mm needles) 240 μL/h.
ND0612-001b	Determine the steady-state plasma concentration of levodopa and carbidopa following continuous SC delivery of ND0612 in healthy volunteers	Phase I, single dose (24-h), single-center, randomized, double-blind, placebo-controlled dose escalation study (*N* = 18)	18 healthy male volunteers (aged 18–40 years old)	• 24-h SC infusion of ND0612 in one infusion site and placebo on the opposite side of the abdomen. • Doses of ND0612 were administered to 3 cohorts of 6 volunteers: ◦ Group A. ND0612 (50/11.7 mg/ml) at a rate of 240 μL/h over 24 h ◦ Group B. ND0612 (60/14 mg/ml) at a rate of 80 μL/h over 8 h and 240 μL/h over 16 h ◦ Group C. ND0612 (60/14 mg/ml) at a rate of 240 μL/h over 24 h administered with entacapone 200 mg five times (every 2 h).
ND0612-002	Establish pharmacokinetic stability of levodopa levels in patients with Parkinson's disease	Single dose, single-center, randomized, double-blind, placebo-controlled, crossover study (N=8)	8 PD patients (Hoehn and Yahr stage <5 during OFF) and receiving optimized levodopa/L-AAAD inhibitor therapy (3–8 doses/day, not exceeding 500 mg/day)	• Patients were randomized (1:1) to ND0612 (60/14 mg/ml at a rate of 0.08 ml/h over 8 h and 0.24 ml/h over 16 h) or placebo in the right side of the abdomen. • SC infusion of ND0612 or placebo was started in the early evening and continued for 24 h. An oral tablet of fixed dose combination immediate-release levodopa/carbidopa/entacapone (100/25/200) was administered at bedtime (~3 h after the start of the infusion) and in the morning on the next day, 15 h after the start of the infusion. Following a 1-week washout period, patients received the other treatment in the left side.
ND0612-003 (35)	Evaluate levodopa pharmacokinetics after repeated dose of ND0612 (14–21 days), safety and tolerability and exploratory efficacy analysis in PD patients	Randomized, double-blind, placebo- controlled, parallel multidose study (*N* = 30), followed by an open-label period (*N* = 16)	30 PD patients (Hoehn and Yahr stage <5 during OFF) on an optimized oral levodopa/L-AAAD inhibitor regimen (≥3 doses per day with ≥3 h between doses) and experiencing ≥2 h/day of OFF time.	• During Period 1, patients were randomized to either ND0612 (60/14 mg/ml at a rate of 0.08 ml/h over 8 h and 0.24 ml/h over 16 h) or matching placebo in addition to their current standard of care (SoC) treatment. Oral levodopa dose reductions were permitted for improving tolerability of the antiparkinsonian drug regimen (such as troublesome dyskinesias), but any changes in the levodopa dosing frequency and interval were discouraged.
				• The first 16 patients who completed Period-1 continued with open-label ND0612 treatment during Period-2. These 16 patients were re-randomized to receive 1 week of ND0612 monotherapy or ND0612 plus oral entacapone while completely eliminating or reducing the oral levodopa/carbidopa dose, based on neurologist's judgement.
ND0612-004	Evaluate levodopa plasma concentrations when ND0612 is infused at two different rates and at two different carbidopa concentrations	Open-label study in which patients received 2 to 3 of 6 possible treatment regimens of ND0612, for up to 3 days in separate, daily, 8-h consecutive infusion periods (*N* = 16)	16 PD patients on optimized oral levodopa/carbidopa doses and experiencing well-defined OFF periods (≥80% of days)	• Patients were assessed at baseline for standard of care (oral levodopa/carbidopa) • Patients were randomized to receive ND0612 at either a low infusion (0.24 ml/h, *n* = 9) or a high infusion (0.64 ml/h, *n* = 7) rate over 8 h. • While the concentration of levodopa (60 mg/ml) remained constant, the carbidopa concentration increased from 7.5 mg/ml on Day 1 to 14 mg/ml on Day 3. • On Day 4, patients received ND0612 (60/14 mg/ml) plus 200 mg entacapone.
ND0612-005	Identify the concentration of infused carbidopa that provides optimal levodopa bioavailability in healthy volunteers	Open-label study in which patients received 1 of 2 different regimens containing 3 different LD/CD ratios (*N* = 20)	Healthy male and female volunteers (30–65 years old)	• ND0612 given at either a low dose (administered through one infusion site, starting at 0.08 ml/h for 8 h and 0.24 ml/h for 16 h) or a high dose (administered through two infusion sites, 0.08 ml/h for 6 h, 0.64 ml/h for 18 h and changing back to 0.08 ml/h for an additional 6 h).
				• While the concentration of levodopa (60 mg/ml) remained constant, the carbidopa concentration tested was 7.5 mg/ml on Day 1, 6 mg/ml on Day 3, and 4 mg/ml on Day 5.

### Pharmacokinetic analyses

During each treatment period, standardized meals with a low content of protein were provided to minimize surges of dietary-derived amino acids, which in some circumstances may compete with levodopa uptake. Serial blood samples for pharmacokinetic analysis of levodopa, carbidopa, and the levodopa metabolite 3-O-Methyldopa (3-OMD) were collected at relevant time points. Plasma levodopa, carbidopa, and 3-OMD concentrations were analyzed using validated liquid chromatography coupled to tandem mass spectrometry (LC/MS/MS) methods.

For levodopa, the lower limit of quantitation (LLOQ) was 50 ng/ml, and the upper limit of quantitation (ULOQ) was 5,000 ng/ml. For carbidopa, the LLOQ was 10 ng/ml and the ULOQ was 1,000 ng/ml. The pharmacokinetic population for each study included all participants who had received at least one dose of ND0612 and had a minimum of 3 quantifiable post-dose plasma concentrations per analyte. Across the studies, pharmacokinetic analysis of the concentration-time data was performed using non-compartmental analysis to obtain the area under the concentration-time curve (AUC) and the maximum observed plasma concentrations (C_max_).

In addition, Study 003 evaluated the time the plasma concentration was above 1,000 ng/ml (T >1,000 ng/ml) (31) and the Fluctuation Index calculated as (C_max_–C_min_)/C_average_ (32). Dose proportionality was assessed in Study 001 using a linear regression analysis of log-transformed levodopa AUC_15 − 24_ and in Study 005 using a power model on log-transformed AUC_0 − inf_, AUC_0 − last_, and C_max_, including terms for dose fitted as a fixed (continuous) effect and participant as a random effect.

### Safety analyses

Safety was assessed in each of the studies through the standard recording of adverse events. In addition, local skin safety was specifically assessed across the studies. Skin assessments typically included the assessment of nodules, hematomas and pain.

## Results

### Levodopa and carbidopa pharmacokinetics

#### Studies 001 and 001b: Demonstration of stable plasma levodopa concentrations following SC delivery with ND0612 in healthy volunteers

**Figure 1:**
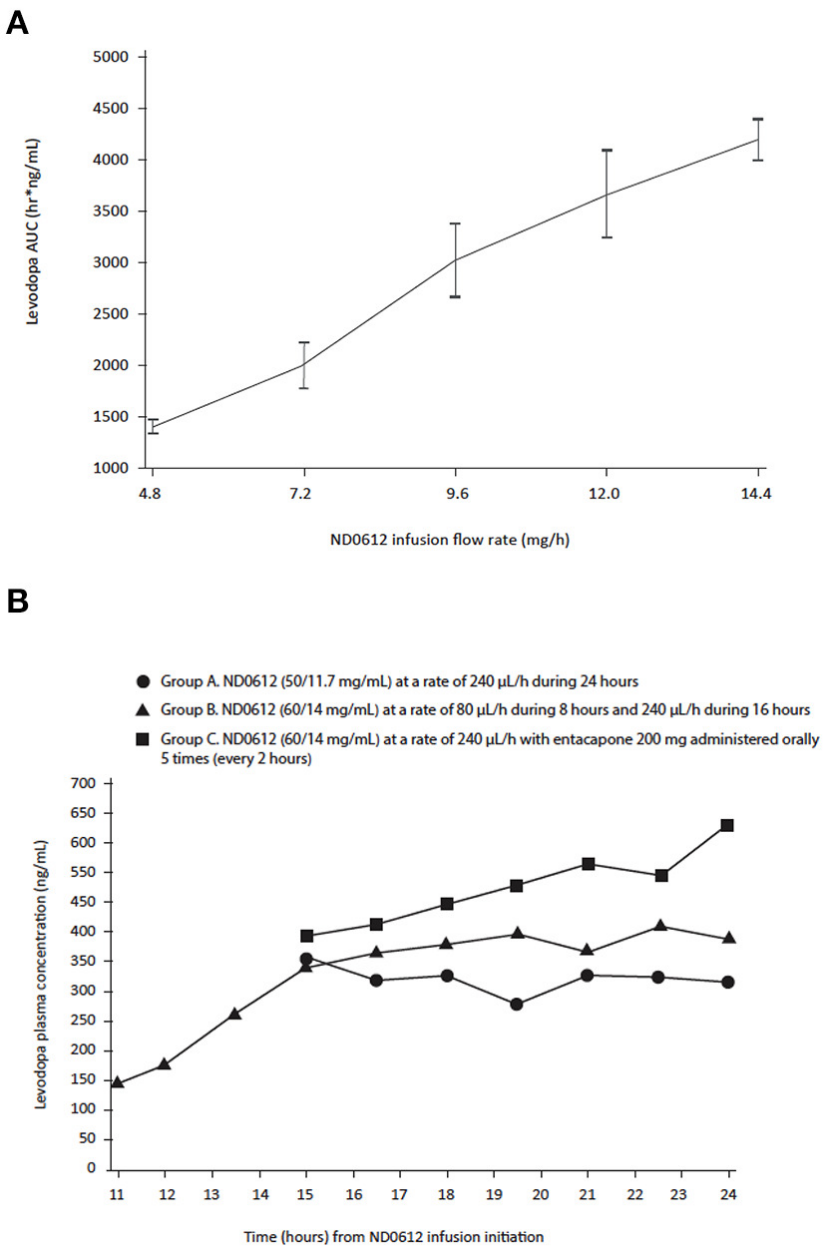
Levodopa **(A)** area under the curve **(B)** concentrations over time following SC infusion with ND0612 in healthy volunteers. **(A)** AUC comparisons between cohorts were made on the 15- to 24-h time period after the start of the infusion **(B)** Blood samples were collected at 11, 12, 13.5, 15 h for Group B only, and 16.5, 18, 19.5, 21, 22.5, and 24 h for all three groups.

Levodopa concentrations generally increased with increasing infusion rates at a nearly dose proportional manner. In Study 001, conducted in healthy volunteers, stable levodopa and carbidopa concentrations were already attained by 15 h (timing of the first blood sample) of a 24-h continuous infusion period, and the study participants maintained these stable concentrations for the remainder of the 24-h infusion period. Mean levodopa AUC_15 − 24_ values ranged from 1,413 h•ng/ml in the 80 μL/h group to 4,199 h•ng/ml in the 240 μL/h group. Linear regression analysis of log-transformed mean AUC_15 − 24_ values demonstrated a slope value of 1.02 (95% CI: 0.92, 1.12) (Figure 1A). Mean AUC values of carbidopa from 15 to 24 h also increased with infusion rate. The average AUC values ranged from 849 h•ng/ml in the 80 μL/h group (Group 1) to 2,201 h•ng/ml in the 240 μL/h group (Group 6). The increase in carbidopa AUC_15 − 24_, as a function of infusion rate, was linear and proportional to rate of infusion demonstrating a slope of 0.92 (95% CI: 0.78, 1.06). Similar results demonstrating carbidopa dose proportionality were obtained for the maximum carbidopa concentration during the 15- to 24-h period and for the mean carbidopa concentration at both 15 and 24 h of the infusion period.

Similar results were seen in Study 001b where, consistent with the results of the previous study, ND0612 administered SC for 24 h achieved clinically relevant plasma concentrations by at least 15 h (Figure 1B). An increase in the concentration of ND0612 (from 50/11.7 to 60/14 mg/ml levodopa/carbidopa) resulted in a corresponding increase in the mean AUC values as would be expected (from 3,070 to 3,677 h•ng/ml). When increasing the infusion rate 3-fold from 80 to 240 μL/h, mean concentrations of levodopa increased over a 4–5-h period and then stabilized. There was a further increase in levodopa concentrations with the addition of entacapone (every 2 h for the last 10 h of the 24-h infusion period) as would be expected with inhibition of COMT activity.

#### Studies 002 and 003: Establishing the pharmacokinetics of levodopa following SC infusion with ND0612 in patients with PD

**Figure 2:**
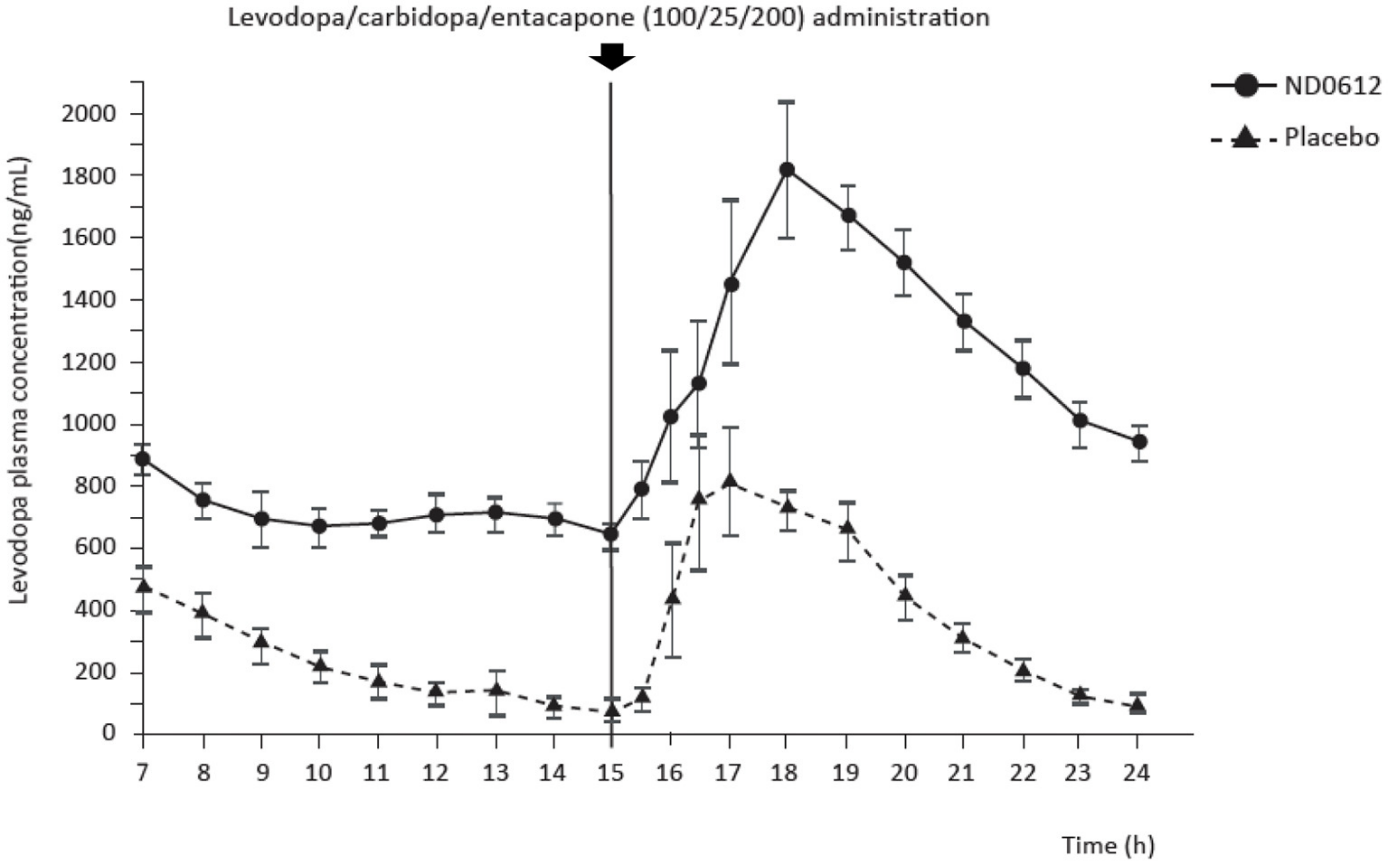
Mean levodopa concentrations over time in patients with Parkinson's disease. Blood sampling began at 7 h post-infusion start. Oral levodopa/carbidopa/entacapone (100/25/200) was taken at 3- and 15-h post-infusion start.

Stable levodopa and carbidopa concentration with ND0612 administration were also demonstrated in patients with PD. In Study 002, where SC infusion of ND0612 started in the early evening and one dose of an oral fixed combination immediate-release levodopa/carbidopa/entacapone [LD/CD/E (100/25/200)] was administered at bedtime (~3 h after the start of the infusion), stable levodopa concentrations were observed from 9 h and stable carbidopa concentrations from 7 h until the morning oral LD/CD/E dose (taken at 15 h). Increases in overall levodopa and carbidopa concentrations (i.e., levodopa and carbidopa from ND0612 + oral LD/CD/E) were observed within 1 h of the morning oral LD/CD/E dose intake with the peak levodopa concentrations occurring at 3 h post-oral LD/CD/E intake; mean levodopa plasma concentrations were sustained ≥25% higher than before administration of the oral LD/CD/E dose for the remainder of the 24-h observation period (Figure 2).

Both studies 002 and 003 used what can be considered a “low” dose of ND0612 (total daily levodopa/carbidopa dose: 270 mg/63 mg). Key pharmacokinetic parameters for levodopa and carbidopa (Cmax, AUC and fluctuation index) are summarized for studies in patients with PD in Tables 2, 3.

**Table 2:** ND0612 levodopa pharmacokinetics in patients with Parkinson's disease.

**Study, *N***	**Baseline demographics**	**Concomitant medications**	***N***	**Regimen**	**C_max_ (ng/ml) mean ±SD**	**T_max_ (h) median (range)**	**AUC (h•ng/ml) mean ±SD**
Study 002	• Age, 66.9 ± 5.3 years • Male sex, 50.0% • White ethnicity, 100% • Duration of PD, 8.1 ± 2.4 years • Duration of motor fluctuations 2.9 ± 2.4 years	Amantadine (63%) Entacapone (38%) Levodopa (100%) Pramipexole (38%) Rasagiline (63%) Ropinirole (38%) Selegiline (38%)	*N* = 8	ND0612 (60/14 mg/ml) at a rate of 0.08 ml/h over 8 h & 0.24 ml/h over 16 h plus Stalevo 100 given at 3- and 15-h post-infusion	2,116 ± 390	3.0 (2.0–5.0)	AUC_7 − 24:_ 17,453 ± 3,206 AUC_15−∞:_ 18,979 ± 4,757
Study 003*	• Age, 63.8 ± 7.4 years • Male sex, 63.2% • White ethnicity, 100% • Duration of PD, 8.6 ± 4.5 years	Amantadine (42%) Pramipexole (11%) Ropinirole (26%) Rasagiline (42%) Selegiline (21%)	*N* = 19	**Period 1:** Standard of care levodopa plus ND0612 (60/14 mg/ml) at a rate of 0.08 ml/h over 8 h & 0.24 ml/h over 16 h	3,515 ± 1,452	5.0 (0.0–10.0)	AUC_0 − 10_: 19,592 ± 9,534
	• Duration of motor fluctuations 5.7 ± 4.2 years	Trihexyphenidyl (11%) Biperiden (5%)	*N* = 8	**Period 2:** ND0612 (60/14 mg/ml) monotherapy at a rate of 0.08 ml/h over 8 h & 0.24 ml/h over 16 h	1,185 ± 864	1.8 (0.0–10.0)	AUC_0 − 10_: 7,297 ± 3,360
			*N* = 8	**Period 2:** ND0612 60/14 mg/ml at a rate of 0.08 ml/h over 8 h & 0.24 ml/h over 16 h plus entacapone 200 mg given every 6 h	2,378 ± 1,553	1.0 (7.0–10.0)	AUC_0 − 10_: 15,017 ± 7,669
Study 004**	• Age, 63.0 ± 7.2 years • Male sex, 75.0%	Amantadine (38%) Trihexyphenidyl (6%)	*N* = 9	ND0612 (60/7.5 mg/ml) at a low rate of 0.24 ml/h over 8 h	618 ± 496	7.0 (6.0–8.0)	AUC _0 − last_: 2.487 ± 874
	• White ethnicity, 100% • Duration of PD, 9.3 ± 3.9 years • Duration of motor fluctuations 5.8 ± 3.9 years	Pramipexole (25%) Ropinirole (25%) Rasagiline (44%) Selegiline (13%) Biperiden (6%)	*N* = 9	ND0612 (60/14 mg/ml) at a low rate of 0.24 ml/h over 8 h	487 ± 104	8.0 (7.0–8.0)	AUC _0 − last_: 2,434 ± 442
			*N* = 8	ND0612 (60/14 mg/ml) at a low rate of 0.24 ml/h over 8 h plus entacapone 200 mg	604 ± 106	8.0 (6.0–8.0)	AUC _0 − last_: 2,923 ± 518
			*N* = 7	ND0612 (60/7.5 mg/ml) at a high rate of 0.64 ml/h over 8 h	1,355 ± 270	8.0 (8.0–8.0)	AUC _0 − last_: 6,466 ± 1,404
			*N* = 7	ND0612 (60/14 mg/ml) at a high rate of 0.64 ml/h over 8 h	1,454 ± 270	8.0 (7.0–8.0)	AUC _0 − last_: 7,549 ± 1,621
			*N* = 7	ND0612 (60/14 mg/ml) at a high rate of 0.64 ml/h over 8 h plus entacapone 200 mg	1,844 ± 382	7.5 (7.0–8.0)	AUC _0 − last_: 8,853 ± 1,558
			*N* = 16	Oral levodopa/carbidopa (baseline)	2,014 ± 861	1.5 (0.5–8.0)	AUC _0 − last_: 6,912 ± 3,077

*Data are for ND0612 are for end of study period. **Modified Fluctuation index (Model's root mean square estimate).

**Table 3:** ND0612 carbidopa pharmacokinetics in patients with Parkinson's disease.

**Study**	***N***	**Regimen**	**C_max_ (ng/ml) mean ±SD**	**T_max_ (h) median (range)**	**AUC (h•ng/ml) mean ±SD**
Study 002	*N* = 8	ND0612 (60/14 mg/ml) monotherapy at a rate of 0.08 ml/h over 8 h & 0.24 ml/h over 16 h	485 ± 102	4.5 (1.5–9.0)	AUC_7 − 24:_ 6,196 ± 1,265
Study 003*	*N* = 19	**Period 1:** Standard of care levodopa plus ND0612 (60/14 mg/ml) at a rate of 0.08 ml/h over 8 h & 0.24 ml/h over 16 h	562 ± 112	6.0 (1.0–10.5)	AUC_0 − last_: 5,082 ± 985
	*N* = 8	**Period 2:** ND0612 (60/14 mg/ml) monotherapy at a rate of 0.08 ml/h over 8 h & 0.24 ml/h over 16 h	538 ± 107	5.0 (0.0–9.0)	AUC_0 − last:_ 4,744 ± 942
	*N* = 8	**Period 2:** ND0612 60/14 mg/ml at a rate of 0.08 ml/h over 8 h & 0.24 ml/h over 16 h plus entacapone 200 mg given every 6 waking h	471± 86	6.0 (4.0–7.0)	AUC_0 − last:_ 4,156 ± 623
Study 004*	*N* = 9	ND0612 (60/7.5 mg/ml) at a low rate of 0.24 ml/h over 8 h	185 ± 37	8.0 (7.0–8.0)	AUC _0 − last_: 834 ± 114
	*N* = 9	ND0612 (60/14 mg/ml) at a low rate of 0.24 ml/h over 8 h	351 ± 94	8.0 (7.0–8.0)	AUC _0 − last_: 1,590 ± 309
	*N* = 8	ND0612 (60/14 mg/ml) at a low rate of 0.24 ml/h over 8 h plus entacapone 200 mg	346 ± 77	8.0 (7.0–8.0)	AUC _0 − last_: 1,633 ± 366
	*N* = 7	ND0612 (60/7.5 mg/ml) at a high rate of 0.64 ml/h over 8 h	477 ± 57	8.0 (7.0–8.0)	AUC _0 − last_: 2,120 ± 286
	*N* = 7	ND0612 (60/14 mg/ml) at a high rate of 0.64 ml/h over 8 h	879 ± 207	8.0 (7.0–8.0)	AUC _0 − last_: 4,173 ± 560
	*N* = 7	ND0612 (60/14 mg/ml) at a high rate of 0.64 ml/h over 8 h plus entacapone 200 mg	835 ± 137	8.0 (6.0–8.0)	AUC _0 − last_: 4,096 ± 587

*Data for ND0612 are for end of study period.

**Figure 3:**
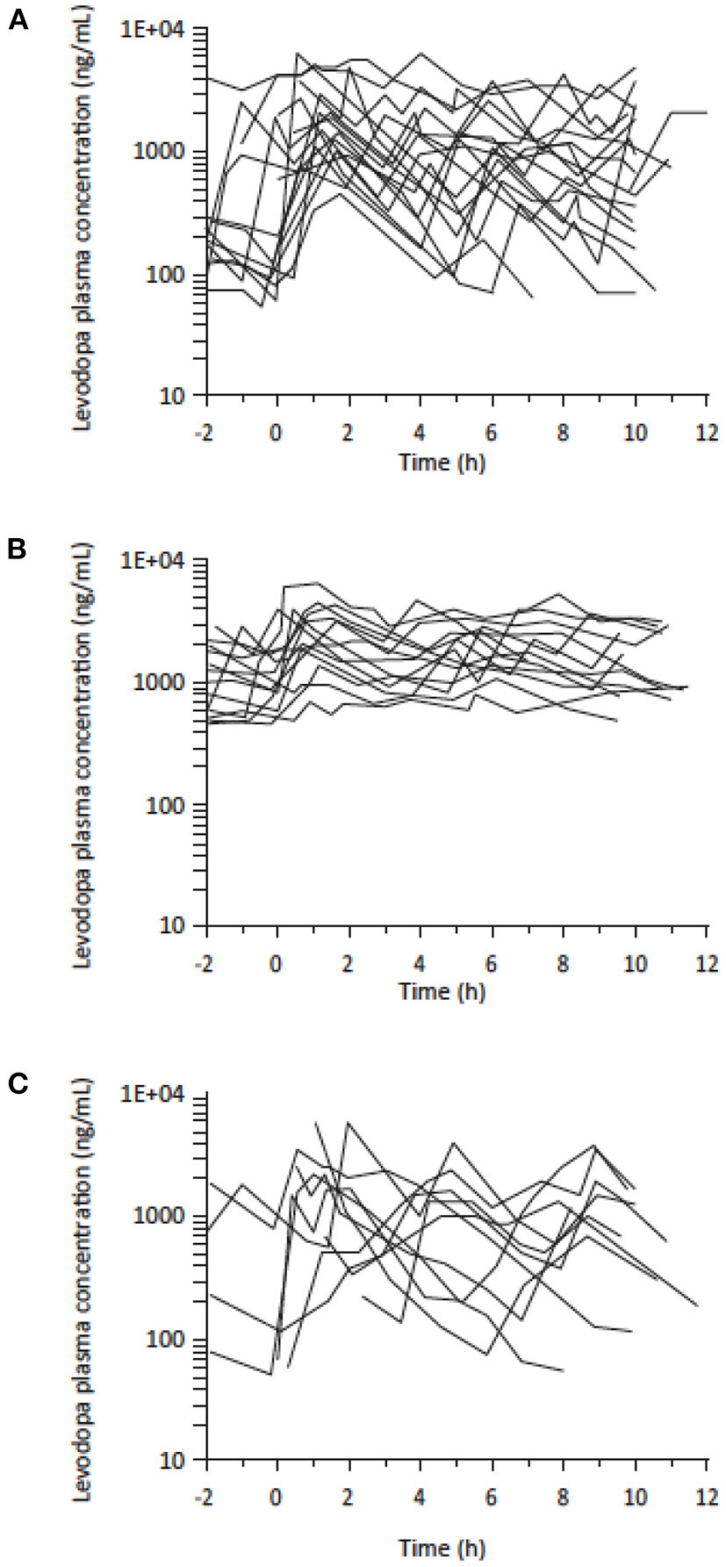
Levodopa plasma levels (logarithmic scale) in patients with PD experiencing motor fluctuations treated with **(A)** standard of care (SoC) levodopa (*N* = 30) **(B)** SoC levodopa plus ND0612 infusion (*N* = 19)* (C) SoC levodopa plus placebo infusion (*N* = 11)*. *Standard of care levodopa plus ND0612 (60/14 mg/ml) or placebo infused at a rate of 0.08 ml/h over 8 h and 0.24 ml/h over 16 h.

Despite the low dose, Study 003 showed that continuous SC administration of ND0612 + SoC oral levodopa/carbidopa ameliorated the variability in levodopa plasma concentrations as compared to placebo infusion + SoC. Patients treated with ND0612 had their plasma levodopa concentrations consistently maintained above a mean of 800 ng/ml, and completely avoided the low trough concentrations observed in the placebo group (Figure 3). There was also a significant increase in the duration that levodopa concentrations above 1,000 ng/ml (mean increase of 4.4 ± 2.2 h in a 10-h period, *p* < 0.0001) which was not apparent in the placebo group (mean of 4.5 h at baseline and at end of Period-1). The increase in mean levodopa exposure achieved with ND0612 + SoC was accompanied by a decreased variability in plasma levodopa concentrations, as evidenced by a decreased Fluctuation Index vs. placebo + SoC (1.6 ± 0.5 vs. 3.1 ± 1.6, respectively, at end of Period-1). The addition of entacapone to the continuous ND0612 SC infusion in Period 2 translated to an increase in mean levodopa AUC compared with monotherapy.

#### Studies 004 and 005: Establishing the optimal carbidopa concentration for ND0612

**Figure 4:**
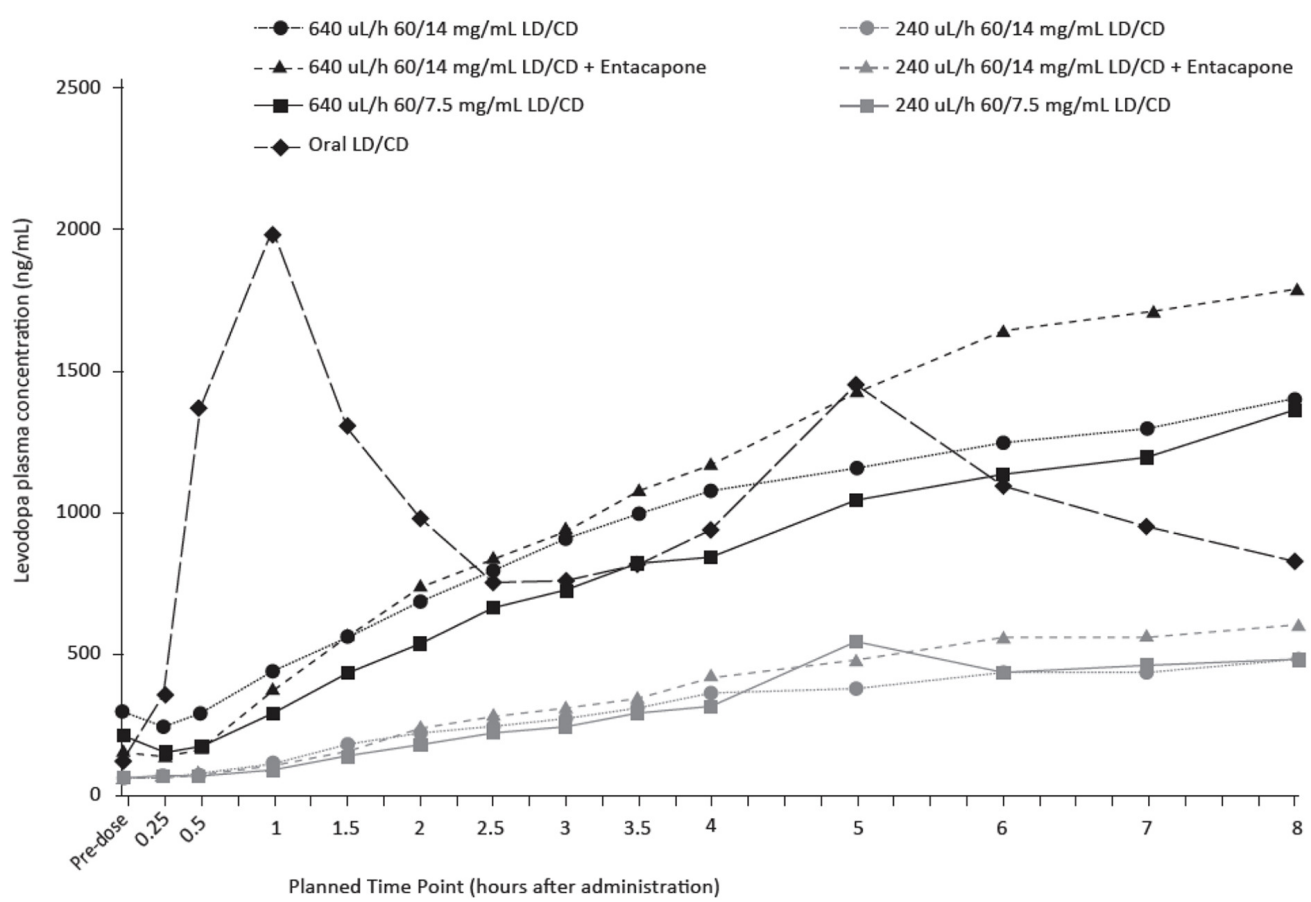
Mean plasma levodopa levels following continuous SC administration of ND0612 over 8 h with varying rates and carbidopa concentrations.

Study 004 explored the impact of two concentrations of carbidopa in the formulation (7.5 and 14 mg/ml, giving a LD/CD ratio of 8:1 and 4:1, respectively) on levodopa concentrations. Both the low and high ND0612 regimens maintained near-constant, therapeutic levodopa plasma concentrations. Levodopa plasma concentrations were dose proportional, with the high dose of 640 μL/h achieving around 3-fold higher plasma concentrations than the low dose 240 μL/h ND0612 regimen, which corresponds to the ~3-fold increase in daily levodopa dose infused. Figure 4 shows the mean plasma levodopa concentrations following continuous SC administration of low and high dose ND0612 with the different carbidopa concentrations in the formulation as well as adding entacapone to the high CD formulations.

The magnitude of the levodopa plasma concentration differences between the two different ND0612 formulations of carbidopa concentration is likely to be not clinically relevant, with both concentrations achieving clinically relevant levodopa concentrations as compared to the oral dosing concentrations. Carbidopa exposure concentrations increased proportionally with carbidopa dose. The addition of entacapone, increased the steady state levodopa concentrations achieved with both regimens.

**Figure 5:**
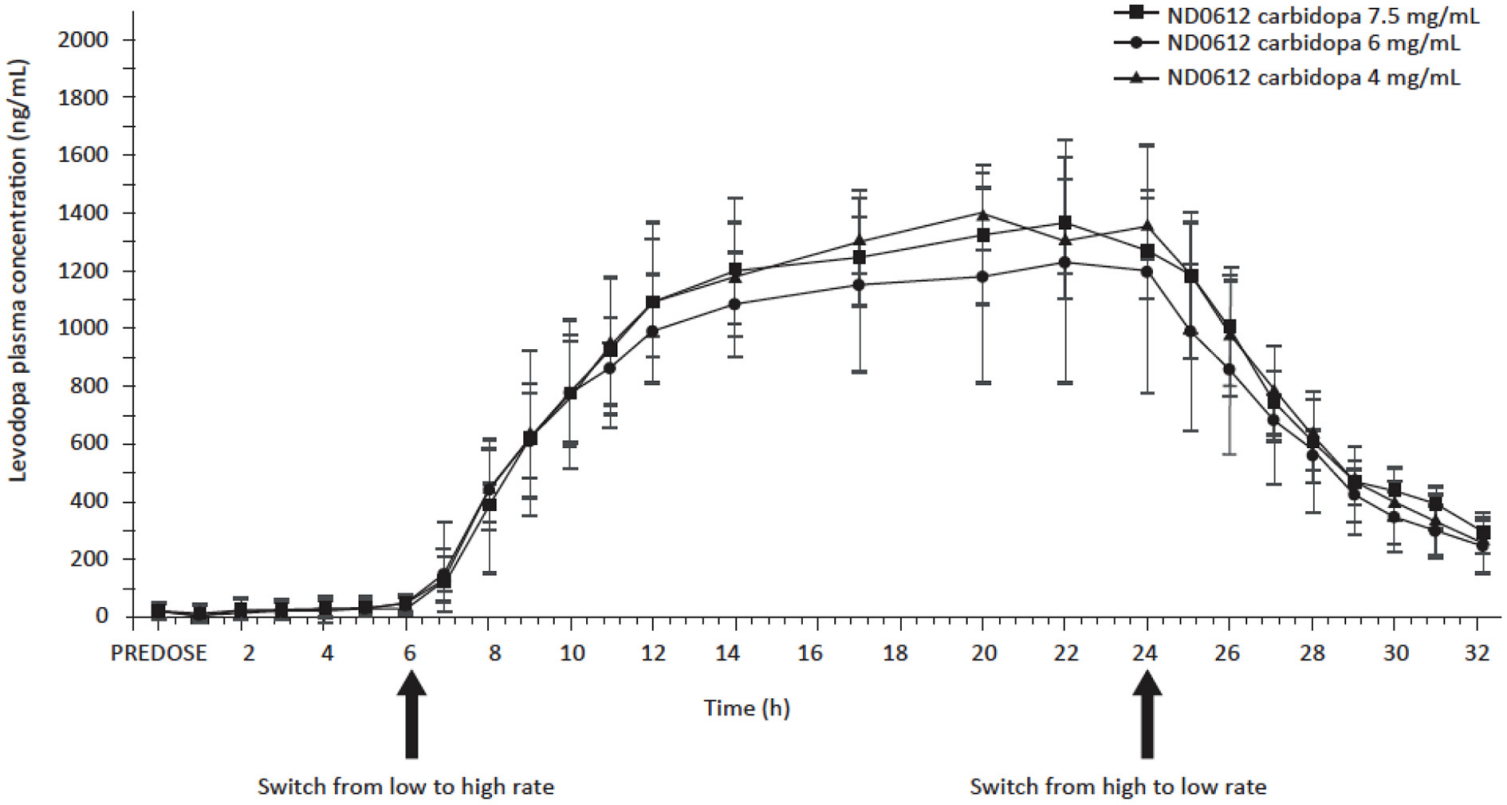
Mean plasma levodopa concentrations following continuous SC administration of ND0612 (high dose) with varying carbidopa concentrations.

The effect of carbidopa concentration was further studied in the 005 study in healthy volunteers where study participants received either low or high rates (doses) of SC ND0612 infusion with three different carbidopa concentrations and ratios from levodopa (7.5 mg/ml, 4:1; 6 mg/ml, 10:1; and 4 mg/ml, 15:1). When administered at the low rate of infusion, mean [95%CI] slope estimates for levodopa exposure on the linear mixed model (log scale) were 0.004 (0.0002, 0.008) and 0.005 (0.001, 0.009) for AUC_0 − last_ and AUC_0 − 24_, respectively. These slope estimates were statistically different from zero (*p* < 0.05), indicating that levodopa AUC rose with increasing carbidopa dose. By contrast, for the high rate of ND0612 infusion, levodopa exposure was relatively unaffected by the carbidopa dose level, with no more than a 5% difference in exposure over the dose range (Figure 5).

For both the high and low infusion rates, carbidopa exposure increased in a dose-proportional manner with respect to increasing carbidopa dose. The 7.5 mg/ml dose of carbidopa consistently resulted in an AUC_0 − inf_ above 2,000 ng•h/ml, which is estimated to provide maximal inhibition of DDC, while lower carbidopa concentrations could potentially compromise levodopa bioavailability (31).

### Safety and tolerability

Across these short-duration studies, there was no consistent pattern of an increase in frequency (number of adverse events), incidence (number of subjects with at least one adverse event), or severity of adverse events (AEs) with increasing infusion rates of ND0612. An overall summary of AEs is given in Supplementary Table 2. All AEs assessed as related to the study drug were mild or moderate in severity, and none of them led to premature treatment discontinuation. The most common AEs were infusion site reactions and/or pain at the infusion site. There were no clinically significant treatment-emergent changes in any clinical laboratory parameters, vital signs, ECG parameters, or physical examination findings.

## Discussion

Subcutaneous infusion of levodopa may provide a well-tolerated and convenient route of continuous levodopa delivery. However, until the development of ND0612, the poor solubility of levodopa precluded this approach. The series of studies described above formed the basis for the final formulation of ND0612 which is now in Phase III of its clinical development. Taken together, the results demonstrate that subcutaneous levodopa infusion achieves stable and sustained plasma concentrations of levodopa as well as dose-proportionality with plasma concentrations increasing with increased infusion rates. Resulting from consistent administration and bypassing the gastrointestinal tract, the stable concentrations of levodopa achieved support ND0612 as a potential parenteral formulation for achieving clinically useful delivery of levodopa for PD patients.

It is generally agreed that plasma levodopa concentration need to be maintained above a certain threshold to achieve sustained relief of PD symptoms. Several investigations have estimated this threshold concentration to be about 1,000 ng/ml (14, 33, 34). In this series of investigations, subcutaneous infusion of “high” doses of ND0612 consistently remained above this threshold and avoided the troughs below the concentrations associated with OFF episodes. Even when lower dosing regimens were trialed in Study 003, continuous SC administration of ND0612 ameliorated both the troughs in levodopa plasma concentrations and the variability in levodopa concentrations (as reflected by the Fluctuation Index vs. placebo). The Fluctuation Index is a measure of the magnitude of rise and fall of levodopa plasma concentrations relative to the average concentration; the lower the Fluctuation Index, the more likely the C_max_ is blunted relative to the trough, thereby minimizing potential C_max_-related adverse effects [including peak-effect dyskinesia (33)]. From these data, it is reasonable to believe that treatment with NDO612 should also ameliorate dyskinesia (since the C_max_ is lower for ND0612 vs. oral formulations). However, a limitation of current and ongoing studies is that they focus on a population of patients primarily suffering from OFF time. In Study 004, both the low and high ND0612 regimens maintained near-constant, therapeutic levodopa plasma concentrations.

Of particular note, the development plan considered that the ideal dosing of carbidopa with levodopa has never been fully established—even for oral formulations. Previous preclinical studies have shown that when carbidopa is continuously delivered subcutaneously, levodopa pharmacokinetics are improved as compared to oral carbidopa administration (35). In part, this may be because there is evidence for inconsistent uptake of oral carbidopa from the gastrointestinal tract. This inconsistency could add to the variability of oral levodopa effect (36). In Study 005 (conducted in 20 healthy volunteers), the concentration of SC infused carbidopa that achieved optimal levodopa bioavailability was found to be 7.5 mg/ml when co-administered with SC levodopa at 60 mg/ml. In other studies, higher carbidopa concentrations did not increase bioavailability of levodopa, though lower concentrations of carbidopa when administered at the “low” infusion rates were found to compromise the levodopa levels—probably due to insufficient inhibition of the DDC enzyme. Thus, taken together, the data generated in this series of investigations found that a levodopa/carbidopa dose ratio of 1:8 would be the most reasonable choice for proceeding with ND0612 in Phase III clinical trials. The choice of the solubilized constituents in ND0612 is now fixed as carbidopa 7.5 mg/ml together with levodopa 60 mg/ml.

The studies presented here also explored augmenting the SC administration of ND0612 with oral levodopa/carbidopa, administered either alone or in combination with the COMT inhibitor entacapone. The supplemental use of these oral drugs produced expected effects on plasma levodopa concentrations and suggested ways that ND0612 could be used in future clinical applications. These results indicate that oral levodopa/carbidopa (with or without a COMT inhibitor) might be a way to lessen the quantity of drug that would otherwise need to be delivered subcutaneously to achieve therapeutic concentrations of carbidopa and levodopa. This ability to predictably complement continuous SC levodopa/carbidopa infusion with oral drugs is likely to provide patients with the flexibility in dosing to optimize their pharmacotherapy (and without the need to rely on full-replacement SC levodopa/carbidopa monotherapy in order to obtain the pharmacokinetic benefits of continuous SC levodopa/carbidopa therapy). The ongoing BouNDless study (NCT04006210) starts with a “conversion period” in which each patient's ND0612 treatment is optimized with supplemental oral levodopa/carbidopa as necessary. Changes to other antiparkinsonian medications are not permitted during all periods of the study.

We have described a series of early development pharmacokinetic studies, which served as a “learning curve” for understanding the peripheral pharmacokinetics of subcutaneously delivered levodopa/carbidopa. At each stage of the program, lessons were learned that impacted subsequent planning and choice of study designs. As such, the studies share the usual early development limitations of being relatively small in size and duration. A population pharmacokinetic model, including data from these studies and additional clinical trials with pharmacokinetic data analyses, is in development. Participants in these studies were predominantly White Caucasians. A pharmacokinetic study in Japanese subjects has already been initiated. Most of the studies tested abdominal infusion placement, whereas in real-world applications, patients will require rotation of sites due to the development of transient infusion site reactions and some will use infusion sites on other areas of the body with significant SC tissue. Recent results from a study comparing the results of abdominal ND0612 infusion with sites in the lower back and thighs confirm bioequivalence from infusion at different sites (35) and peer reviewed data will be published separately. While all the studies included safety observations, the studies were too short to be of relevance and the 1-year ND0612 safety data recently reported by Poewe et al. (37) are much more informative. We note that the low dosing regimens in the very earliest clinical development studies were originally investigated to understand the feasibility of small “patch pumps” in a less severe patient population. Ultimately, it was decided to prioritize the development of the “high” dosing regimen (up to 720/90 mg/day) infused over 16 and/or 24 h because of the significant unmet needs of patients with poorly controlled motor fluctuations. Therefore, this is the chosen treatment regimen under investigation in ongoing clinical trials (37, 38). While it could be argued that a key limitation of the development program is the lack of information on the pharmacokinetics of ND0612 monotherapy in patients with PD, it is pertinent to note that ND0612 is not being developed as a “complete” dopamine replacement therapy because patients with motor fluctuations typically require relatively high levodopa doses which would be impractical to deliver as a subcutaneous infusion therapy due to skin tolerability. Rather, ND0612 is intended as the next evolution in levodopa delivery for patients with Parkinson's disease. Future studies may continue the development of lower dose ND0612 regimens for patients who require less levodopa, potentially, with small patch-pump style devices.

In summary, ND0612 is under development as a minimally invasive drug-device combination to provide continuous subcutaneous delivery of levodopa/carbidopa for patients with PD experiencing motor fluctuations. Taken together, this series of clinical pharmacokinetic investigations have demonstrated that ND0612, administered continuously with a levodopa concentration of 60 mg/ml together with carbidopa 7.5 mg/ml, and complemented with oral levodopa/carbidopa, is suitable for administration for up to 24 h in patients with PD. This formulation has been shown to be generally safe and well-tolerated in an open-label, 12-month study of over 200 patients with PD (37) and we await the results of the ongoing Phase III study which will establish whether the favorable pharmacokinetic profile of ND0612 translates into clinical efficacy.

## Data availability statement

The original contributions presented in the study are included in the article/Supplementary material, further inquiries can be directed to the corresponding author/s.

## Ethics statement

The studies involving human participants were reviewed and approved by Institutional Review Boards at the participating sites approved the study protocol, consent forms, and associated amendments. The patients/participants provided their written informed consent to participate in this study.

## Author contributions

PL, FS, DA, YC, and NG were investigators involved in the early ND0612 clinical development program. IP provided pharmacokinetic analysis of the concentration-time data and interpretation of results. LA was involved in trial coordination and interpretation of results. PL, LA, and RC contributed to the first draft equally. All authors provided critical review of the manuscript and approved the final version of the article.

## Conflict of interest

Author PL reports advisory roles for Abide, Acorda Therapeutics, Adamas, Biogen, Cavion, Denali, Intec Pharma, Jazz Pharmaceuticals, Lundbeck, Neurocrine, Mitsubishi NeuroDerm Ltd., Prexton, Revance, Sage, and US WorldMeds; lecture fees from US WorldMeds, Acorda, American Academy of Neurology, and Kyowa Hakko Kirin. Research grant support from Abide, Acorda, Amneal, Lundbeck, Michael J. Fox Foundation for Parkinson's Research, Mitsubishi NeuroDerm Ltd., Parkinson Study Group; National Institute of Neurological Disorders and Stroke, Pharma 2B, Revance, Hoffmann-La Roche; Sunovion, and Sun Pharma. Author FS reports honoraria and consulting fees from Britannia Pharmaceuticals, GlaxoSmithKline, Boehringer Ingelheim, Lundbeck, Orion, NeuroDerm, Novartis, Teva, Pfizer and Zambon. Authors YC and IP report consultancy for NeuroDerm. Authors LA and RC are employed by NeuroDerm. Author NG serves as consultant to Sionara, NeuroDerm, Pharma2B, Denali, Neuron23 and Abbvie, Sanofi-Genzyme and Biogen. He receives royalties from Lysosomal Therapeutics (LTI) and payment for lectures at Abbvie, Sanofi-Genzyme and Movement Disorder Society. He received research support from the Michael J. Fox Foundation, the National Parkinson Foundation, the European Union and the Israel Science Foundation as well as from Teva NNE program, Biogen and Ionis. He receives support from the Sieratzki Family Foundation and the Aufzien Academic Center in Tel-Aviv University. The remaining author declares that the research was conducted in the absence of any commercial or financial relationships that could be construed as a potential conflict of interest.

## Publisher's note

All claims expressed in this article are solely those of the authors and do not necessarily represent those of their affiliated organizations, or those of the publisher, the editors and the reviewers. Any product that may be evaluated in this article, or claim that may be made by its manufacturer, is not guaranteed or endorsed by the publisher.
