# Supplementary material for: The pharmacokinetics of continuous subcutaneous levodopa/carbidopa infusion: Findings from the ND0612 clinical development program
Source: Front Neurol. 2022 Nov 10;13:1036068. doi: 10.3389/fneur.2022.1036068 (PMC9686322; doi:10.3389/fneur.2022.1036068)
Supplement: Supplementary file 1 [file Data_Sheet_1.pdf]

Supplemental Appendix

Study ND0612-001

A Phase I, Single-dose, Single-center, Randomized, Double-blind, Placebo-controlled Dose Escalation Study Evaluating Safety, Tolerability, and LD Plasma Concentration Following Administration of SC Continuously delivered LD/CD Solution (ND0612) in Healthy Volunteers

Study design and Population

Study ND0612-001 was a single-dose, randomized, double-blind, placebo-controlled dose-escalation study in healthy male participants. The main objectives of the study were to characterize the safety, tolerability, and exposure levels of LD and CD following sequential increases in the SC infusion rate of ND0612 LD/CD 60/14 mg/mL (LD:CD = 4:1 ratio) from one cohort to the next in healthy adult participants.

Each participant was administered ND0612 and placebo (saline) continuously for 24 hours, at the same rate at two parallel infusion sites (randomized to left (L) side vs right (R) side) in the abdomen using the Accu-Chek® Spirit Combo pump

Participants were enrolled into one of the six dosing cohorts

ND0612-001: Dosing Cohorts for ND0612 (LD/CD 60/14 mg/mL in solution) or Placebo (saline solution)

Cohort (N)	SC Infusion Rate (µL/h) (10 mm needle)	Amount of Drug Administered (mg) in 24 Hours		
		LD	CD	LD:CD Ratio
1 (N=6)	80	115.2	26.9	4:1
2 (N=6)	120	172.8	40.3	4:1
3 (N=6)	160	230.4	53.8	4:1
4 (N=6)	200	288	67.2	4:1
5 (N=6)	200[1]	288	67.2	4:1
6 (N=6)	240	345.6	80.6	4:1

[1]6mm needle

The study population consisted of 36 healthy male volunteers, with mean ± SD years of age (24.3 ± 3.3), and weight (72.9 ± 9.5 kg).

Study ND0612-001b

A Phase Ib, Single Dose, Single-center, Randomized, Double-blind, Placebo-controlled Dose Escalation Study Evaluating Safety, Tolerability, and LD Plasma Concentration Following Administration of SC Continuously delivered LD/CD Solution (ND0612) in Healthy Volunteers.

Study Design and Population

Study ND0612 was a randomized, single-dose, double-blind, placebo-controlled dose escalation study in healthy male participants to determine the safety, tolerability, and exposure levels of LD and CD following continuous SC administration of ND0612 for 24 hours, at two different concentrations and three different treatment regimens. Each participant was administered ND0612 and placebo (saline) continuously for 24 hours, at two parallel infusion sites (randomized to left (L) side vs right (R) side) in the abdomen using the CRONO Five pump.

ND0612-001b: Dosing Regimens of ND0612

Treatment Group/Cohort	Dosing Regimen	Total Dose (mg)	
		LD	CD
**A**	LD/CD 50/11.7 mg/mL (4:1 LD:CD) at a rate of 240 µL/h for 24 h	288.0	67.4
**B**	LC/CD 60/14 mg/mL (4:1 LD:CD) at a rate of 80 µL/h for 8 h and then 240 µL/h for 16 h.	268.8	62.8
**C**	LC/CD 60/14 mg/mL (4:1 LD:CD) at a rate of 240 µL/h for 24 h + oral entacapone 200 mg, administered orally, 5 times during the day	345.6	80.6

LD = levodopa; CD = carbidopa

A total of 18 healthy male participants were enrolled (6 per treatment group) in the study, with mean ± SD years of age (25.8 ± 4.9), and weight (74.3 ± 9.1 kg).

Study ND0612-002

A Phase I/IIa, Single dose, Single-centre, Randomized, Crossover, Double-blind, Placebo-controlled Study Evaluating Safety, Tolerability, and LD Plasma Concentration Following Administration of SC Continuously Delivered LD/CD Solution (ND0612 in PD Patients).

Study Design and Population

Study ND0612-002 was designed to evaluate the safety, tolerability and steady-state exposure levels of LD and CD following SC administration of ND0612 (LD/CD 60/14 mg/mL; 4:1 LD:CD) with 2 doses of levodopa/carbidopa/entacapone (LD/CD/E 100/25/200 mg tablets) compared with SC administration of placebo (saline) with 2 doses of STALEVO 100in patients with PD.

In separate treatments, ND0612 LD/CD 60/14 mg/mL or placebo were infused SC at a rate of 80 µL/h for the initial 8 hours (amount of LD/CD = 38.4/9 mg) and then at 240 µL/h for 16 hours (amount of LD/CD = 230.4/53.8 mg), for a total 24-hour LD/CD dose of 268.8/62.8 mg. An oral tablet of LD/CD/E was administered at bedtime (approximately 3 hours after the start of the infusion) and in the morning on the next day, 15 hours after the start of the infusion.

Patients were randomized to receive either Treatment A (ND0612 + LD/CD/E) or Treatment B (placebo + LD/CD/E) during period 1 and the opposite treatment following a 1-week washout period. PK endpoints were the steady-state plasma concentration of LD and CD following SC continuous delivery and the LD PK profile following oral LD/CD with and without ND0612.

A total of 8 patients with PD (4 male and 4 female) were evaluated in the study. The mean (SD) age and BMI of the study participants were 66.9 ± 5.3 years, and 31.5 ± 6.1 kg/m^2 respectively.

Study ND0612-003

A Phase IIa Multicenter Randomized Double-blind, Placebo-controlled Study followed by an Open-label Period to Evaluate the Safety, Tolerability and LD Pharmacokinetics in LD-treated PD Patients with Motor Fluctuations, Administered with Repeated Continuous SC Infusions of ND0612

Study Design and Population

Study ND0612-003 was a two-periods, multicenter study consisting of a randomized, double-blind, placebo-controlled comparator phase (Period 1) and a follow-up open-label extension phase (Period 2) with ND0612 (LD/CD 60/14 mg/mL, LD:CD 4.3:1) in PD patients with motor fluctuations being treated with oral LC/CD therapy. The objectives of the study were to determine the PK of LD derived from ND0612 on top of the standard of care, to evaluate the safety and tolerability of SC ND0612 following a repeated-dose treatment regimen of 14-to-21 days, and exploratory efficacy outcomes. Further assessment was done to evaluate the effect of entacapone on LD PK derived from ND0612. During Period 1 of the study, 30 patients with PD and motor fluctuations were randomized 2:1 (ND0612:placebo), with 19 of the participants receiving ND0612 and 11 receiving placebo, with all 30 participants being maintained on their baseline oral LD/CD treatment regimens. Both ND0612 and placebo (saline) were administered by continuous SC infusion for 14-days using an SC delivery pump (CRONO-ND). During every 24-hour cycle, the study drug was initially administered at a slow rate of SC infusion (80 μL/h) over 8 hours, representing a night-time LD/CD dose of 38.4/9 mg, which was followed by a higher rate of infusion (240 μL/h) over 16-hours, representing a daytime LD/CD dose of 230.4/53.8 mg, for a total daily LD/CD dose of 270/63 mg.

During Period 2 of the study, 16 participants who had completed Period 1 elected to continue receiving ND0612 as an open-label extension treatment, with half of the participants receiving ND0612 in combination with entacapone (200 mg/day administered 3 times) for an additional 7-day period. Oral LD treatment was to be completely stopped unless needed by the patient.

Blood samples for PK analysis were obtained on Study Days 2 and 15 from all participants during Period 1 and on Study Day 22 from participants who continued in Period 2.

A total of 30 male and female LD-treated PD patients with motor fluctuations were enrolled in the study. The BMI of the study population ranged from 18 to 27.9 kg/m^2.

Study ND0612-004

Study Title

A Multi-center, Open-Label, Randomized, Dose-Finding Study Testing the Safety, Tolerability, and Pharmacokinetics (PK) of ND0612, a Liquid Formulation of LD/CD Delivered as a Continuous SC Infusion in PD Patients Treated with LD

Study Design and Population

This was a 2-center, open-label, randomized, dose-finding study in which two formulations of ND0612 (60/7.5 mg and 60/14 mg) as well as concomitant treatment with entacapone were studied in Low and High LD doses (115 mg and 307 mg, respectively), delivered as a continuous SC infusion via an infusion pump (CRONO-ND) for three 8-hour consecutive infusion periods. The objectives of the study were to 1) assess the safety and tolerability and PK of 6 different dosing regimens of ND0612 in PD patients with well-defined morning "OFF" period and good response to LD therapy, 2) determine the effects of CD dose and rate of infusion on the PK variability of LD vs. intermittent oral dosing of LD/CD therapy, and 3) evaluate exploratory efficacy outcome.

Participants were randomized to either Low or High dose of ND0612 (LD 115 mg administered via 1 infusion site and 307 mg administered via 2 infusion sites, respectively). In each group three 8-hour treatment regimens were studied consequently on Days 3, 4 and 5 as shown in **Error! Reference source not found.** table. ND0612 formulations with LD/CD ratio of 60/7.5 mg (8:1) or 60/14 mg (4:1) were studied in both groups on Day 3 and Day 4, respectively. Entacapone combination treatment with ND0612 60/14 mg (4:1) regimen was optional on Day 5.

ND0612-004 Dosing Regimens

<table>
<tr><th>Study Group</th><th>Treatment Regimen Number</th><th>Study Day</th><th>ND0612 Formulation
(LD/CD, mg/mL)</th><th>Infusion Rate
(mL/h)</th><th>No. of Infusion Sites</th><th>Infusion Duration
(hours)</th><th>Total LD/CD Dose/8 h
(mg)</th><th>Oral Entacapone</th></tr>
<tr><td rowspan="3">1
ND0612L
Low LD Dose</td><td>1</td><td>3</td><td>60/7.5</td><td rowspan="3">0.24</td><td rowspan="3">1</td><td rowspan="6">8</td><td>115/14</td><td>No</td></tr>
<tr><td>2</td><td>4</td><td>60/14</td><td>115/27</td><td>No</td></tr>
<tr><td>3 [a]</td><td>5</td><td>60/14</td><td>115/27</td><td>200 mg every 4 h</td></tr>
<tr><td rowspan="3">2
ND0612H
High LD Dose</td><td>4</td><td>3</td><td>60/7.5</td><td rowspan="3">0.64 [b]</td><td rowspan="3">2</td><td>307/38</td><td>No</td></tr>
<tr><td>5</td><td>4</td><td>60/14</td><td>307/72</td><td>No</td></tr>
<tr><td>6 [a]</td><td>5</td><td>60/14</td><td>307/72</td><td>200 mg every 4 h</td></tr>
<tr><td colspan="9">h = hours; LD/CD = levodopa/carbidopa</td></tr>
<tr><td colspan="9">[a] = Optional
[b] = The value corresponds to the cumulative infusion rate through both sites</td></tr>
</table>

Male and female (3:1) Caucasian patients with PD and with well-defined morning "OFF" time and a good response to LD were enrolled in the study. The mean (± SD) age and BMI of the participants were 63 ± 7.2 years (range 49 to 73 years) and 29 ± 5.1 kg/m^2 (range 22.6 to 41.0), respectively.

Study ND0612-005

An Open-Label Study in Healthy Male and Female Participants to Identify the Concentration of CD that Provides Optimal Bioavailability of a Concomitant Fixed Concentration of LD infused SC via a Pump System

Study Design and Population

This study investigated the PK and safety of formulations with 3 different ratios of LD:CD – Dose 1 (8:1), Dose 2 (10:1), and Dose 3 (15:1) – administered, via one infusion site with CRONO pump with a single syringe driver system for the 269mg LD arm (low dose ND0612) and via CRONO TWIN pump with a dual syringe driver system for the 720mg LD arm (high dose ND0612).
