# Supplementary material for: The pharmacokinetics of continuous subcutaneous levodopa/carbidopa infusion: Findings from the ND0612 clinical development program
Source: Front Neurol. 2022 Nov 10;13:1036068. doi: 10.3389/fneur.2022.1036068 (PMC9686322; doi:10.3389/fneur.2022.1036068)
Supplement: Supplementary file 2 [file Data_Sheet_2.pdf]

Supplementary Table: Treatment Emergent Adverse Events in ND0612 Levodopa pharmacokinetics studies

Number of Subjects (%) with treatment-emergent adverse event (TEAE)	ND0612-001 N=36	ND0612-001b			ND0612-002		ND0612-003				ND0612-004		ND0612—005a	
							Period 1		Period 2					
		Group A N=6	Group B N=6	Group C N=6	ND0612 N=8	Placebo N=8	ND0612 N=19	Placebo N=11	ND0612 without entacapone N=8	ND0612 with entacapone N=8	Low infusion rate N=9	High infusion rate N=7	ND0612L N=8	ND0612H N=13
**Any TEAE**	34 (94.4)	4 (66.7)	6 (100)	5 (83.3)	6 (75.0)	3 (37.5)	9 (47.4)	3 (27.3)	5 (62.5)	3 (37.5)	5 (55.6)	3 (42.9)	7 (87.5)	9 (69.2)
**Any serious TEAE**	0	0	0	0	0	1 (12.5) (not related)	1 (5) (possibly related)[2]	0	1 (13) (not related)	1 (13) (not related)	0	0	0	0
**Administration site AE[1] (local skin safety)**	32 (88.9)	3 (50)	6 (100)	4 (66.7)	5 (62.5)	0	1 (5)	0	0	0	*	*	5 (62.5)	6 (46.2)

[1] Captured by AE reporting under the MedDRA System Organ Class *'General disorders and administration site conditions'*

[2] Syncope reported as SAE (with seriousness criterion = hospitalisation, one day) in a patient with a medical history of orthostatic hypotension, who had forgotten to take his anti-orthostatic hypotension medication on the SAE onset day.

*In Study 004, local skin safety was assessed with the Draize score and its components (erythema and edema) as well as pain score, pruritus score and presence of a nodule and staining across the study.

Number (%) of subjects with positive findings for local infusion site evaluations	Low infusion rate N=9	High infusion rate N=7
**Positive Draize score**	6 (67%)	7 (100%)
**Erythema and eschar**	5 (56%)	6 (86%)
**Edema**	3 (33%)	5 (71%)
**Pruritus**	2 (22%)	2 (29%)
**Presence of Nodules**	8 (89%)	7 (100%)
**Staining**	5 (56%)	6 (86%)
**Infusion site pain**	2 (22%)	6 (86%)
